# Comprehensive Investigation of GRF Transcription Factors and Associated Responses to Drought Stress in Oat (*Avena sativa*)

**DOI:** 10.3390/plants15010160

**Published:** 2026-01-05

**Authors:** Shirui Xu, Xiajie Ji, Fumeng Sai, Mingchuan Ma, Zhang Liu, Lijun Zhang, Longlong Liu

**Affiliations:** 1 Center for Agricultural Genetic Resources Research, Shanxi Agricultural University, Taiyuan 030031, China; 2 Houji Laboratory of Shanxi Province, Taiyuan 030031, China; 3 Institute of Agricultural Information and Economics, Hebei Academy of Agriculture and Forestry Sciences, Shijiazhuang 050051, China; 4 College of Agriculture, Shanxi Agricultural University, Jinzhong 030801, China

**Keywords:** growth-regulating factor, *Avena sativa*, genome-wide analysis, expression patterns, drought stress

## Abstract

Growth-regulating factors (GRFs) are plant-specific transcription factors that play important roles in plant growth and development. However, no systematic analysis of *GRF* genes has been reported in oat (*Avena sativa*). In this study, we conducted a comprehensive characterization of the *GRF* gene family in oat, including their physicochemical properties, chromosomal distribution, phylogenetic relationships, gene structure, conserved domains, promoter *cis*-elements, duplication events, and drought-responsive expression. In total, 28 *GRF* genes were identified in oat. Phylogenetic analysis classified them into two main groups, which could be further subdivided into five subgroups. Gene structure and conserved motif analyses revealed that *AsGRF* genes are largely group-specific and relatively highly conserved within each subgroup. Segmental duplication has been the primary driver of *AsGRF* gene family expansion, and these genes have undergone strong purifying selection during evolution. Transcriptomic analysis identified 13 *AsGRF* genes expressed under drought stress. Subsequent qRT-PCR analysis revealed that six of these genes were significantly up-regulated. Notably, *AsGRF3* showed the highest expression level, was localized to the nucleus, and lacked transcriptional self-activation activity. In conclusion, this study provides a comprehensive analysis of the *AsGRF* gene family and serves as a valuable reference for further functional characterization of these genes in drought stress responses in oat.

## 1. Introduction

Growth-regulating factors (GRFs) constitute a highly conserved, plant-specific family of transcription factors that regulate multiple processes in plant growth and development. They coordinate organogenesis and plant architecture in leaves, stems, roots, flowers, and seeds, and mediate responses to biotic and abiotic stresses [1,2]. GRFs are characterized by two highly conserved N-terminal domains: QLQ (Gln, Leu, and Gln) and WRC (Trp, Arg, and Cys) [2]. The QLQ domain contains a canonical glutamine-leucine-glutamine motif (QX_3_LX_2_Q), along with conserved aromatic/hydrophobic and acidic residues. This domain primarily mediates protein-protein interactions. The WRC domain harbors a conserved C_3_H-type zinc finger motif (CX_9_CX_10_CX_2_H) and a cluster of basic residues (rich in arginine and lysine), both essential for nuclear localization and DNA-binding activity [3,4,5]. The C-terminal region of GRF proteins exhibits low sequence conservation and functions as a transactivation domain. Although highly variable in both length and amino acid composition, this region contains short amino acid motifs: TQL (Thr, Gln, and Leu), GGPL (Gly, Gly, Pro, and Leu), and FFD (Phe, Phe, and Asp) [6,7].

As sessile organisms, plants are constantly exposed to various environmental stresses. Drought stress is a major constraint that severely restricts plant growth and development, ultimately leading to substantial reductions in crop yield and quality. In the context of global climate change, extreme drought events are projected to become more frequent and intense. This trend is expected to reduce freshwater availability by up to 50% by 2050 and lead to more extensive and prolonged drought conditions in many regions worldwide [8,9]. To mitigate drought stress, plants have evolved a sophisticated regulatory network. *GRF* genes function as key transcriptional regulators of the drought response. For instance, in *Arabidopsis*, *GRF1* and *GRF3* target multiple drought-responsive genes to modulate drought adaptation [1]. *AtGRF7* regulates drought stress by modulating *DREB2A* expression [10]. In *Medicago truncatula* and *M.* *sativa*, *MtGRF2*-*MsGRF2* and *MtGRF6*-*MsGRF6* are upregulated under drought stress, suggesting they contribute to drought tolerance [11]. Conversely, silencing *CsGRF04* in sweet orange enhances drought resistance [12]. Additionally, the *miR396-GRF* module modulates plant growth under drought stress through *miR396*-mediated repression of *GRF* expression [13]. *SimiR396d* negatively regulates *SiGRF1* in foxtail millet, thereby enhancing drought tolerance and promoting root growth [14].

Oat (*Avena sativa* L.), an allohexaploid species in the Poaceae family, possesses superior nutritional profiles compared with many other cereal grains, characterized by high levels of lipids, proteins, and essential amino acids [15]. It serves not only as an excellent dietary resource but also as a high-quality forage crop [16]. Notably, oats contain bioactive compounds such as β-glucans and avenanthramides, which contribute to various health benefits including lowering cholesterol levels, anti-inflammatory effects, vasodilation, cytoprotection, and anti-carcinogenic activity [17,18]. Owing to these advantages, oats have gained increasing popularity for human consumption. However, oat growth and productivity are severely constrained by drought in many regions worldwide [19]. Therefore, elucidating the drought response mechanisms in oat is crucial for enhancing productivity under water-limited conditions. To date, several oat gene families have been identified as responsive to drought stress. For instance, members of the *AsMYB2R* subfamily (*AsMYB2R039*, *AsMYB2R043*, and *AsMYB2R045*), the *AsBZR* gene family (*AsBZR12*) and the alternative oxidase family (*AsAOX5*) have been demonstrated to play important roles under drought conditions [20,21,22]. However, the role of *GRF* gene family in mediating drought adaptation in oats remains unexplored.

The *GRF* gene family has been systematically identified in a wide range of plant species, including cereals such as wheat [23,24], maize [25], rice [26], sorghum [5,27], and foxtail millet [25,28]; legumes such as soybean [29], alfalfa [30], chickpea, and pigeonpea [31]; as well as other species including potato [32], sugarcane [33], and ginseng [34]. However, this family has not yet been comprehensively characterized in oat. Therefore, this study aimed to (1) perform a genome-wide identification and characterization of the *GRF* gene family in oat, and (2) elucidate their expression patterns and potential functions under drought stress. *AsGRF* genes were systematically analyzed for physicochemical properties, chromosome locations, phylogenetic relationships, exon-intron structures, conserved domains, promoter *cis*-elements, and gene duplication events. Furthermore, we analyzed transcriptome data and performed quantitative reverse transcription PCR (qRT-PCR) to investigate the expression patterns of *AsGRF* genes under drought stress. These analyses revealed that *AsGRF3* was the most highly upregulated gene in roots. Based on this finding, we examined the subcellular localization and transcriptional self-activation activity of *AsGRF3*. This study provides valuable molecular information on the roles of GRF transcription factors in oat adaptation to drought stress and lays a foundation for future functional studies of *AsGRF* genes.

## 2. Results

### 2.1. Identification of GRF Gene Family in A. sativa

To identify the *GRF* gene family in *A. sativa*, we employed a combined approach using BLASTP searches and conserved domain (WRC/QLQ) HMM screening. After deduplication and validation of domain architecture, a total of 28 *AsGRF* genes were identified. These genes were systematically named *AsGRF1* to *AsGRF28* according to their physical positions on the chromosomes (Table 1).

The physicochemical properties of AsGRF proteins in *A. sativa* were analyzed. The proteins ranged from 220 (AsGRF15 and AsGRF21) to 588 (AsGRF26) amino acids in length, with predicted molecular weights of 22.96–62.71 kDa (mean = 40.22 kDa). AsGRF15 (22.96 kDa) and AsGRF26 (62.71 kDa) represent the lowest and highest molecular weight, respectively. Theoretical isoelectric points (pI) spanned from 5.02 (AsGRF3) to 10.26 (AsGRF4), with an average of 8.38. Most AsGRF proteins exhibited pI values above 7, classifying them as basic; however, four members (AsGRF3, AsGRF9, AsGRF11, and AsGRF26) displayed acidic pI values (<7). All AsGRF proteins displayed instability indices above 40, indicating that they are likely unstable. Their aliphatic indices varied from 46.98 (AsGRF28) to 74.45 (AsGRF1). The negative grand average of hydropathicity (GRAVY) values for all proteins confirmed their hydrophilic nature. These results indicate that AsGRF proteins are likely unstable and hydrophilic proteins. Conserved domain analysis predicted lengths of 33–34 amino acids for the QLQ domain and 40–41 amino acids for the WRC domain. Furthermore, subcellular localization predicted that all AsGRF proteins are localized to the nucleus.

### 2.2. Chromosomal Distribution and Phylogenetic Analysis of AsGRF Genes

**Figure 1:**
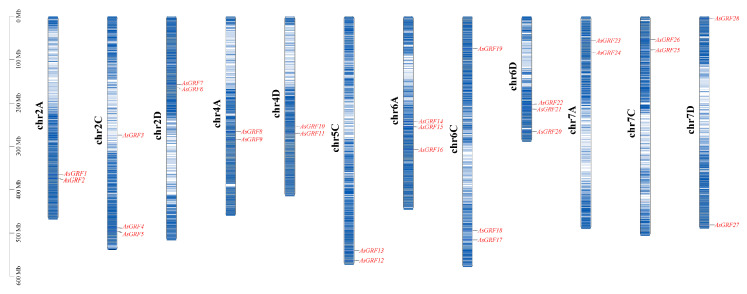
Chromosome localization of *AsGRF* genes.

Chromosomal mapping revealed that the 28 *AsGRF* genes were distributed across 12 chromosomes (Figure 1). Two genes were located on each of the following chromosomes: 2A (*AsGRF1* and *AsGRF2*), 2D (*AsGRF6* and *AsGRF7*), 4A (*AsGRF8* and *AsGRF9*), 4D (*AsGRF10* and *AsGRF11*), 5C (*AsGRF12* and *AsGRF13*), 7A (*AsGRF23* and *AsGRF24*), 7C (*AsGRF25* and *AsGRF26*), and 7D (*AsGRF27* and *AsGRF28*). In contrast, three genes were found on each of chromosomes 2C (*AsGRF3*, *AsGRF4* and *AsGRF5*), 6A (*AsGRF14*, *AsGRF15* and *AsGRF16*), 6C (*AsGRF17*, *AsGRF18* and *AsGRF19*), 6D (*AsGRF20*, *AsGRF21* and *AsGRF22*). Overall, the *AsGRF* genes were relatively evenly distributed among the A, C, and D sub-genomes, with 9, 10, and 9 members, respectively.

**Figure 2:**
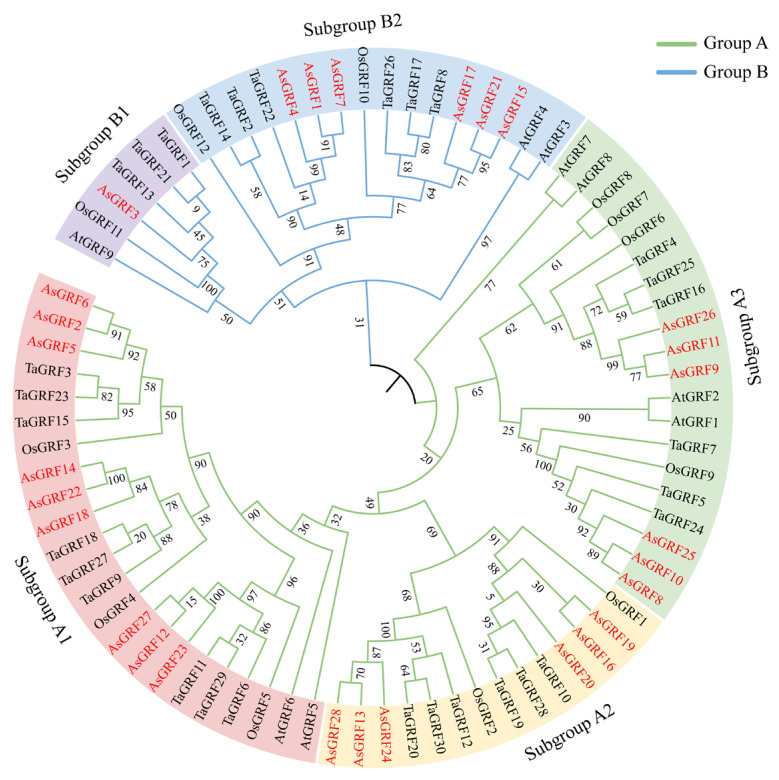
Phylogenetic analysis of the *GRF* family. A phylogenetic tree was constructed using GRF protein sequences from *A. sativa*, *A. thaliana*, *O. sativa*, and *T. aestivum*. The AsGRF proteins are highlighted in red, and distinct colors represent different clades.

**Figure 3:**
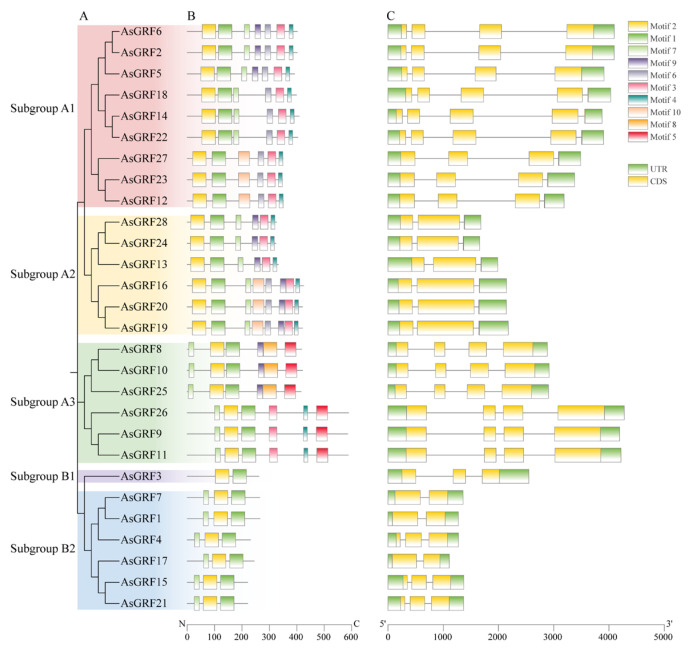
Phylogenetic tree, conserved motif, and gene structure of *AsGRF* gene family in oat. (**A**) The phylogenetic tree of AsGRF proteins. (**B**) The conserved motifs of AsGRFs. (**C**) Exon-intron structures of *AsGRF* genes.

To investigate the evolutionary relationships among *AsGRF* genes, we constructed a phylogenetic tree using GRF protein sequences from *A. sativa* (28), *A. thaliana* (9), *O. sativa* (12), and *Triticum aestivum* (30). Based on phylogenetic analysis of protein sequences, the 79 GRF proteins were classified into two main groups, A and B. Group A was further divided into three subgroups (A1–A3), and Group B into two subgroups (B1 and B2) (Figure 2 and Figure 3A). Subgroup A1 contained the largest number of GRFs, with 9 AsGRFs, 9 TaGRFs, 3 OsGRFs, and 2 AtGRFs. Subgroup A2 contained 6 AsGRFs, 6 TaGRFs, and 2 OsGRFs, with no AtGRF members. Subgroup A3 consisted of 6 AsGRFs, 6 TaGRFs, 4 OsGRFs, and 4 AtGRFs. Within Group B, subgroup B2 included 6 AsGRFs, 6 TaGRFs, 2 OsGRFs, and 2 AtGRFs. In contrast, subgroup B1 was the smallest, comprising a single AsGRF, 3 TaGRFs, one OsGRF, and one AtGRF. In contrast, the number of AsGRFs and TaGRFs was identical in all other subgroups. Notably, all AsGRF proteins consistently clustered within the same phylogenetic clades as TaGRF proteins, indicating their close evolutionary relationship.

### 2.3. Gene Structure and Motif Distribution of AsGRF Genes

To further explore the structural characteristics of the *AsGRF* gene family, we analyzed conserved domains and exon-intron organizations in the context of their phylogenetic relationships. Analysis of conserved motifs showed that all AsGRF proteins contained motif 1 and motif 2, and most also possessed motif 7, except for AsGRF3, AsGRF12, AsGRF23, and AsGRF27. Furthermore, all subgroup A1 members contained motifs 3, 4, and 6, and all subgroup A2 members contained motifs 3, 4, and 9. Interestingly, motif 5 was exclusive to subgroup A3 (Figure 3B and Figure S1). The exon-intron structures were largely group-specific. Genes in subgroup A2 contained three exons and two introns, while those in subgroup A3 possessed four exons and three introns. Most subgroup A1 genes also exhibited four exons and three introns, except for *AsGRF14*, *AsGRF18*, and *AsGRF22*, which contained five exons and four introns. In subgroup B2, *AsGRF4*, *AsGRF15*, and *AsGRF21* shared a structure of three exons and two introns, whereas *AsGRF1*, *AsGRF7*, and *AsGRF17* had only two exons and one intron (Figure 3C). Collectively, these analyses demonstrate that the *AsGRF* genes are structurally similar and relatively highly conserved within their respective phylogenetic clades.

### 2.4. Cis-Acting Element Prediction of AsGRF Genes

**Figure 4:**
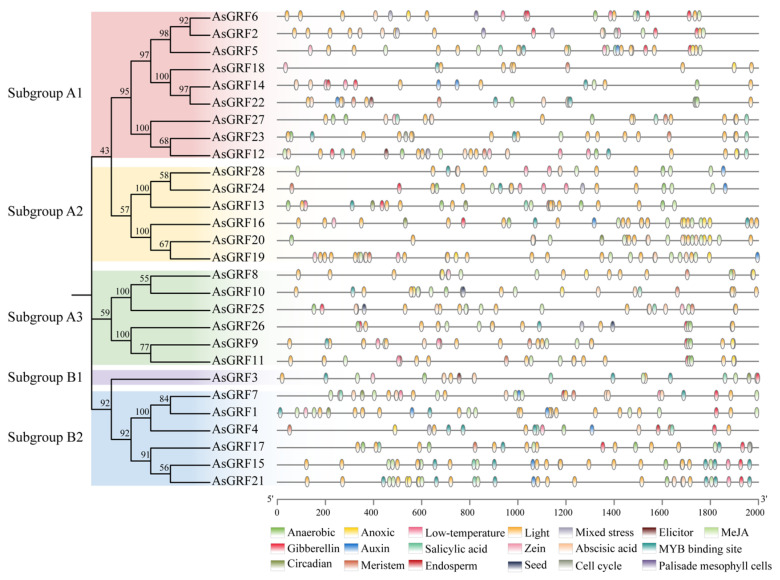
Identification of *cis*-acting elements in *AsGRF* gene promoters. Different elements are indicated by colored boxes.

To functionally characterize the potential regulatory mechanisms of *AsGRF* genes, we identified *cis*-acting elements in their promoter regions (Figure 4 and Figure S2). All promoters contained light-responsive elements. Abscisic acid-responsive elements and MYB binding sites were present in 27 and 22 promoters, respectively. Other elements associated with hormone responses (e.g., auxin and gibberellin) and stress responses (e.g., low temperature) were also detected. The total number of regulatory elements varied across genes, ranging from 10 in *AsGRF18* to 41 in *AsGRF19*. The distinct composition and quantity of *cis*-regulatory elements suggest potential functional divergence in the regulation and biological roles among *AsGRF* family members.

### 2.5. Duplication and Synteny Analysis of AsGRF Genes

**Figure 5:**
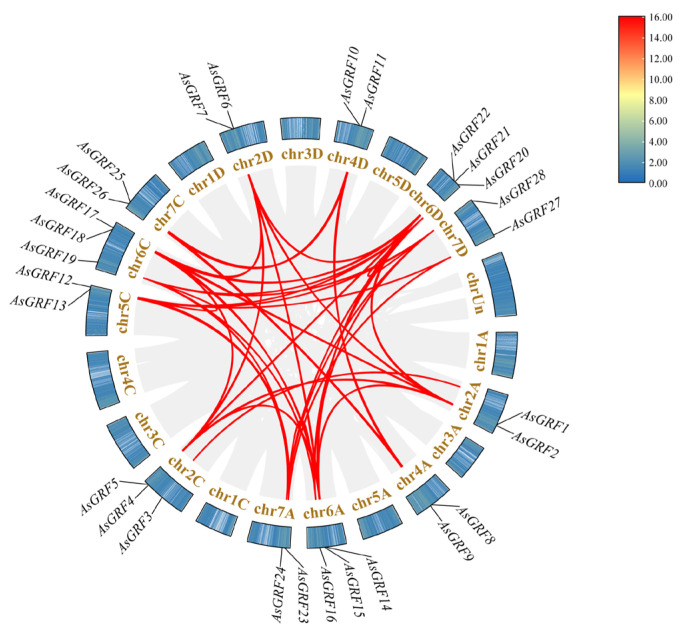
Duplication events of the *AsGRF* genes in oat. Collinearity relationships are shown in gray, with duplication events of *AsGRF* genes highlighted in red.

To elucidate the evolutionary dynamics of the *GRF* gene family in oat, we conducted a comprehensive analysis of gene duplication events (Figure 5, Table S1). A total of 50 segmental duplication events were identified, 49 of which involved *AsGRF* genes at both ends, while one pair consisted of an *AsGRF* gene and a non-*AsGRF* gene. Notably, several *AsGRF* genes exhibited multiple duplication events, no tandem duplication events were detected, suggesting that segmental duplication has been the primary driver of *AsGRF* gene family expansion. Furthermore, *Ka*/*Ks* analysis of duplicated pairs revealed ratios consistently less than 1, ranging from 0.06 to 0.82, indicating that *AsGRF* genes have undergone strong purifying selection during evolution (Table S2).

**Figure 6:**
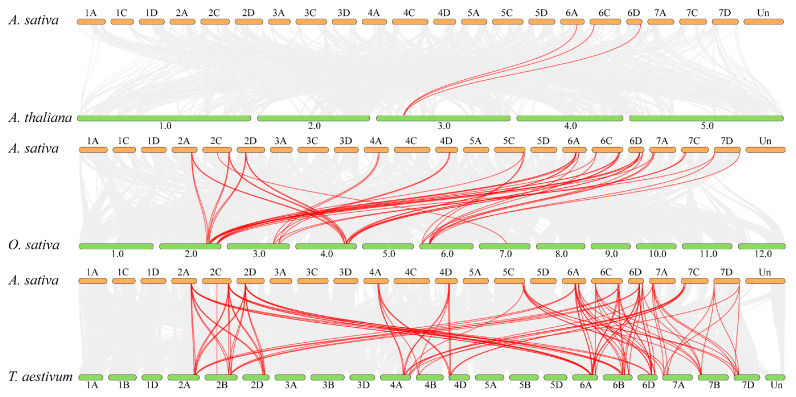
Synteny analysis of *GRF* gene family in *A. sativa*, *A. thaliana*, *O. sativa*, and *T. aestivum*. Gray lines in the background indicate the collinear gene pairs within *A. sativa* and other plant genomes, and the red lines highlight the syntenic *GRF* gene pairs.

To further understand the evolutionary mechanisms of the *AsGRF* genes, we performed synteny analysis between *A. sativa* and three species: *A. thaliana*, *O. sativa*, and *T. aestivum* (Figure 6). The analysis revealed 3 orthologous gene pairs with *A. thaliana* (Table S3), 46 with *O. sativa* (Table S4) and 101 with *T. aestivum* (Table S5). Specifically, 28 *AsGRF* genes showed collinearity with 10 *OsGRF* and 27 *TaGRF* genes, while only three *AsGRF* genes were collinear relationship with *AtGRF5* in *A. thaliana*.

### 2.6. Expression Pattern Analysis of AsGRF Genes Under Drought Stress

**Figure 7:**
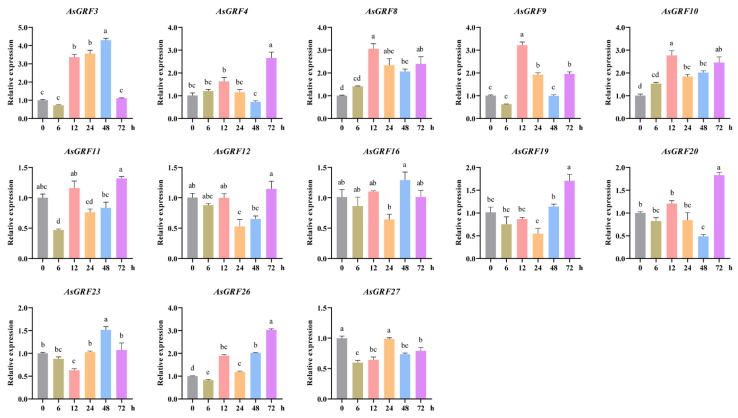
Expression patterns of *AsGRF* genes in the roots of *A. sativa* under drought stress. The error bars represent ± SEMs of three independent experiments. Different lowercase letters indicate statistically significant differences (*p* < 0.05) as determined by one-way ANOVA with Tukey’s test.

To investigate the potential roles of *AsGRF* genes in drought stress, we analyzed their expression profiles in a publicly available oat transcriptome dataset. Transcripts per million (TPM) values showed that 15 of the 28 *AsGRF* genes had TPM < 0.5, indicating no expression under drought stress (Table S6). The remaining 13 detectably expressed *AsGRF* genes were selected for subsequent qRT-PCR validation at six time points after drought treatment (0, 6, 12, 24, 48, and 72 h) (Figure S3). The qRT-PCR results confirmed that six genes (*AsGRF3*, *AsGRF4*, *AsGRF8*, *AsGRF9*, *AsGRF10*, and *AsGRF19*) were significantly up-regulated, while four genes (*AsGRF11*, *AsGRF12*, *AsGRF16*, and *AsGRF27*) were significantly down-regulated at one or more time points. Three genes (*AsGRF20*, *AsGRF23*, and *AsGRF26*) displayed dynamic expression patterns with fluctuations between up-regulation and down-regulation across the time course (Figure 7). These differential expression patterns suggest that *AsGRF* genes may play important and potentially specialized roles in oat drought stress response.

### 2.7. Subcellular Localization and Transcriptional Self-Activation Activity Analysis of AsGRF3 Protein

**Figure 8:**
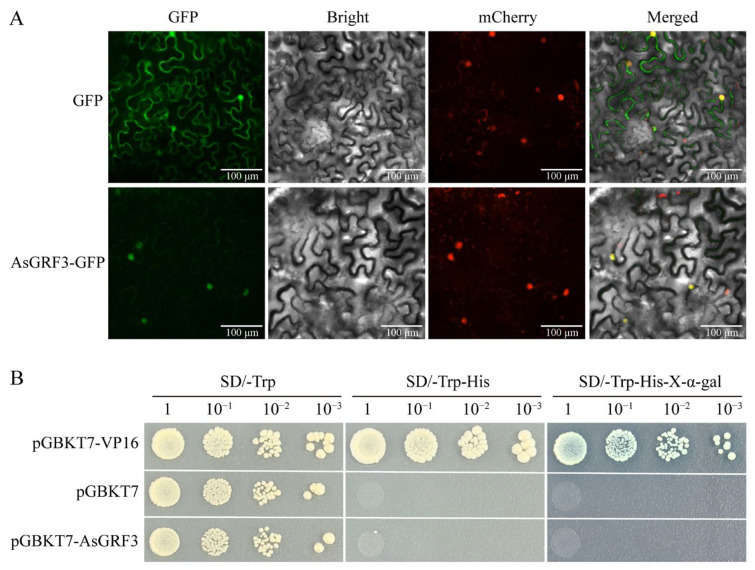
Analysis of subcellular localization and transcriptional self-activation activity of AsGRF3. (**A**) Subcellular localization of AsGRF3. Scale bars correspond to 100 μm. (**B**) Transcriptional self-activation activity analysis of AsGRF3 in yeast.

Given that *AsGRF3* exhibited the highest expression level under drought stress, we propose that it plays a crucial role in oat drought response. We investigated its subcellular localization and transcriptional self-activation activity to confirm its function as a transcription factor. Transient expression of an AsGRF3-GFP fusion protein in *Nicotiana benthamiana* leaves showed that the fluorescence was exclusively localized to the nucleus, confirming AsGRF3 to be a nuclear protein (Figure 8A).

To investigate the transcriptional self-activation activity of AsGRF3, the coding region was cloned into the yeast expression vector *pGBKT7* and transformed into yeast cells. The yeast cells containing pGBKT7-AsGRF3 did not grow on selective medium and remained colorless in X-α-Gal assays (Figure 8B). These results demonstrate that AsGRF3 lacks transcriptional self-activation activity in yeast.

## 3. Discussion

### 3.1. Identification and Physicochemical Characterization of AsGRF Genes

GRF transcription factors are plant-specific regulators that play pivotal roles in growth, development, and adaptation to multiple environmental stresses. For instance, in rice, *OsGRF6* enhances resistance to bacterial blight and grain yield, while *OsGRF7* modulates salt tolerance and grain size [35,36]. In maize, *ZmGRF1* functions downstream of *ZmMPK3* to regulate salt stress responses [37]. Additionally, *HcGRF3* and *HcGRF21* from *Hibiscus cannabinus*, confer enhanced salt and drought tolerance when overexpressed [38]. Driven by the functional importance of GRF proteins, the expanding genomic resources have enabled the systematic identification of *GRF* families across species. Current reports indicate the presence of 9 *GRFs* in *A. thaliana* [39], 12 in *O. sativa* [26], 10 in *Setaria italica* [25,28], 17 in *Zea mays* [25], and 30 in *T. aestivum*. In this study, we identified 28 *GRF* genes in allohexaploid oat. The smaller gene number in oat compared to the 30 *GRFs* in wheat may be attributed to its smaller genome size (∼11 Gb in oat vs. ∼15 Gb in wheat) [40,41], despite both species having undergone whole-genome duplication events during evolution. Among the 28 AsGRF members, 24 exhibited pI above 7, which classifies them as basic proteins. An instability index above 40 suggests protein instability [42]. Notably, all members are predicted to be unstable, as their instability indices exceed the threshold. Furthermore, the GRAVY values for all members were negative. Taken together, these computational predictions indicate that AsGRF proteins are likely to be unstable and hydrophilic.

### 3.2. Evolutionary Phylogeny and Structural Characterization of AsGRF Genes

The phylogenetic tree revealed that GRF proteins from the same species were distributed across different clades (Figure 2). Such a pattern is characteristic of expanded gene families, in which paralogs generated by ancient duplication events have diverged and consequently cluster with orthologs from other species rather than with each other [43,44]. Notably, AsGRF proteins clustered closely with TaGRF proteins to form species-pair-specific subclades. These combined oat-wheat units then shared a common ancestral node with OsGRF proteins, forming larger monophyletic groups. This nested topology indicates that the principal diversification of these *GRF* genes occurred after the rice lineage split but before the oat-wheat divergence from their common ancestor. Indeed, all AsGRFs were clustered with TaGRFs, demonstrating their conserved orthologous relationships. A similar clustering pattern was observed between SbGRFs and OsGRFs [5].

Conserved domain analysis confirmed that all AsGRF proteins contain two characteristic motifs, motif 1 and motif 2, which correspond to the WRC and QLQ domains, respectively. Consistently, the amino acid sequence alignment of 28 AsGRFs showed that all AsGRFs contained the conserved WRC domain and QLQ domain (Figure S4). Notably, AsGRF3 contained only these two conserved motifs. This universal presence indicates their fundamental role in the core function of the AsGRF proteins. In contrast, motif 5 was restricted to subgroup A3 members, while motif 8 was exclusively detected in AsGRF8, AsGRF10, and AsGRF25. This specific distribution suggests that these motifs may confer distinct functional or regulatory properties. Analysis of exon-intron structures revealed considerable diversity in exon number, ranging from two to five across the *AsGRF* genes. Interestingly, this range is consistent with reports for the *TaGRF* and *SitGRF* genes [23,24,28]. The preservation of a similar structural spectrum across these species suggests that the fundamental architecture of *GRF* genes is evolutionarily relatively conserved. Subgroup A2, subgroup B1 and three genes in subgroup B2 contained three exons, while subgroup A3 and six genes in subgroup A1 had four. By contrast, *AsGRF14*, *AsGRF18*, and *AsGRF22* from subgroup A1 possessed five exons, and *AsGRF1*, *AsGRF7*, and *AsGRF17* from subgroup B2 contained only two. Promoter *cis*-acting element prediction revealed substantial divergence in the types and quantities of regulatory elements among *AsGRF* genes. For instance, *AsGRF12* contained 13 distinct element categories, whereas *AsGRF18* possessed only five. Although all *AsGRF* promoters harbored light-responsive elements, their abundance varied significantly, ranging from 3 in *AsGRF4* to 17 in *AsGRF19* (Figure S2). Likewise, all *MdGRF* promoters also possessed light-response elements [45]. This pronounced heterogeneity in *cis*-regulatory architecture suggests potential functional diversification among *AsGRF* members during plant growth and stress responses.

### 3.3. Evolutionary Dynamics of the GRF Gene Family in Oat

To investigate the genetic relationships and evolutionary history of *GRF* genes, we performed a comprehensive analysis including phylogenetic reconstruction and collinearity assessment among *A. sativa*, *A. thaliana*, *O. sativa*, and *T. aestivum*. In the phylogenetic tree, all AsGRF proteins clustered closely with their orthologs from wheat, indicating a high degree of sequence similarity. Consistent with this, synteny analysis revealed the highest number (101) of collinear gene pairs between oat and wheat. A total of 24 *AsGRF* genes were each found to have at least three syntenic orthologs among wheat *GRF* genes, further supporting a strong genomic synteny between the two species. Collectively, these results demonstrate that *AsGRF* genes are more closely related to homologs in *T. aestivum* than to those in *A. thaliana* or *O. sativa*, underscoring the close evolutionary relationship between oat and wheat. Additionally, we calculated the *Ka*/*Ks* ratios for both duplicated *AsGRF* gene pairs and orthologous gene pairs between oat and rice or wheat. In evolutionary biology, a *Ka*/*Ks* ratio less than 1 indicates that a gene has undergone purifying selection [20,46]. Our analysis revealed that all syntenic gene pairs between *AsGRF* and *OsGRF* exhibited *Ka*/*Ks* ratios of less than 1. Among the *AsGRF*-*TaGRF* orthologous pairs, *Ka*/*Ks* ratios could not be calculated for two pairs, one pair exhibited a ratio greater than 1, and all others had ratios less than 1 (Table S7). Similarly, all duplicated *AsGRF* gene pairs also displayed *Ka*/*Ks* ratios of less than 1. These results collectively indicate that the *GRF* genes in these species have predominantly undergone purifying selection during evolution.

### 3.4. Nuclear Localization and Absence of Self-Activation in AsGRF3

Subcellular localization confirmed the nuclear localization of AsGRF3, fulfilling a key prerequisite for its function as a transcription factor. In yeast transactivation assays, however, AsGRF3 displayed no self-activation activity. This characteristic has been previously observed in PheGRF9a of moso bamboo and in the japonica rice OjGRF1, OjGRF4, OjGRF6 and OjGRF7 [47,48]. It is noteworthy that the latter four OjGRFs demonstrate significant transcriptional activity in dual-luciferase assays using rice protoplasts, indicating that the lack of self-activation in yeast does not preclude transactivation in planta [48]. While this study characterized the drought-responsive expression profile of *AsGRF3*, its true transcriptional capacity and functional role during drought stress await further investigation.

## 4. Materials and Methods

### 4.1. Identification of GRF Gene Family Members in A. sativa

The *GRF* gene family in *A. sativa* was identified through a combined computational approach using Hidden Markov Model (HMM) profiling and BLASTP program of BLAST+ (v2.16.0+) analysis. The genome and protein sequences of *A. sativa* were retrieved from the GrainGenes database (https://wheat.pw.usda.gov/GG3/content/avena-sang-download, accessed on 22 October 2024) [49]. BLASTP analysis was performed using known GRF protein sequences from *A. thaliana* (9 sequences; https://www.arabidopsis.org/, accessed on 11 November 2024) [39] and *O. sativa* (12 sequences; https://rice.uga.edu/, accessed on 11 November 2024) (Table S8) [26] as queries, with an *e*-value cutoff of 1 × 10^−5^. Simultaneously, For HMM-based screening, the oat protein sequences were searched against the conserved domain profiles of QLQ (PF08880) and WRC (PF08879) [2]. The overlapping sequences from both HMM and BLASTP searches were merged, and redundant entries were removed while retaining the longest transcript per gene locus. The resulting non-redundant sequences were further validated for the presence of both QLQ and WRC domains through domain analysis using SMART (https://smart.embl.de/, accessed on 20 November 2024), Pfam (http://pfam.xfam.org/, accessed on 20 November 2024), and the Conserved Domain Database of the National Center for Biotechnology Information (NCBI-CDD) (https://www.ncbi.nlm.nih.gov/Structure/cdd/wrpsb.cgi, accessed on 20 November 2024). Genes encoding proteins with both complete domains were confirmed as candidate *AsGRF* genes. The physicochemical properties of the AsGRF proteins were analyzed using the Protein Parameter Calculator in TBtools (v2.376) [50]. The subcellular localization of the AsGRF proteins was predicted using WoLF PSORT (https://wolfpsort.hgc.jp/, accessed on 20 January 2025). The boundaries of the QLQ and WRC domains in the AsGRF proteins were determined using the NCBI-CDD.

### 4.2. Conserved Domain, Gene Structure, Chromosome Localization, and Cis-Acting Analysis

The conserved domains and motifs of AsGRF proteins were performed using the MEME suite (https://meme-suite.org/meme/tools/meme, accessed on 20 January 2025) with the maximum motif number set to 10 [51]. Gene structures, including intron-exon organization, were determined based on the *A. sativa* genome annotation and visualized using the Visualize Gene Structure module in TBtools. The chromosomal locations of *AsGRF* genes were mapped with the Gene Location Visualization tool in TBtools according to the reference genome coordinates. To identify *cis*-acting regulatory elements, a 2000 bp genomic region upstream of the translation start site of each *AsGRF* gene was extracted and analyzed via the PlantCARE database (https://bioinformatics.psb.ugent.be/webtools/plantcare/html/, accessed on 20 January 2025).

### 4.3. Phylogenetic Tree

A phylogenetic tree was performed using the GRF protein sequences from *A. thaliana*, *O. sativa*, *T. aestivum*, and *A. sativa* (Table S8). Multiple sequence alignment was performed with MEGA 7.0, and a maximum likelihood tree was constructed with 1000 bootstrap replicates. The final tree was visualized and landscaped using the online tool Tree Of Life (iTOL) (https://itol.embl.de/, accessed on 12 October 2025).

### 4.4. Duplication, Synteny, and Ka/Ks Analysis of AsGRF Genes

Genomic collinearity analysis of *GRF* genes across *A. sativa*, *A. thaliana*, *O. sativa*, and *T. aestivum* was conducted using the One Step MCScanX module in TBtools with an *e*-value threshold of 1 × 10^−10^. This analysis identified both interspecies orthologs and intraspecific duplication events within the *AsGRF* gene family. For syntenic gene pairs, evolutionary parameters including the nonsynonymous substitution rate (*Ka*), synonymous substitution rate (*Ks*), and *Ka*/*Ks* ratio were calculated using the Simple *Ka*/*Ks* Calculator in TBtools.

### 4.5. Plant Materials and Treatment

The experiment used *A. sativa* cv. Pinyan No. 8, chosen due to its demonstrated drought tolerance in our field studies and its overall agronomic superiority, and was provided by the Center for Agricultural Genetic Resources Research at Shanxi Agricultural University. Seeds were sterilized with sodium hypochlorite (1.0%) for 20 min and then rinsed three times with sterile water. After germination, seedlings were cultivated in modified Hoagland nutrient solution (Coolaber, Beijing, China). The nutrient solution contained the following components per liter: 945 mg Ca(NO_3_)_2_·4H_2_O, 506 mg KNO_3_, 80 mg NH_4_NO_3_, 136 mg KH_2_PO_4_, 241 mg MgSO_4_, 36.7 mg FeNaEDTA, 0.83 mg KI, 6.2 mg H_3_BO_3_, 16.9 mg MnSO_4_·H_2_O, 8.6 mg ZnSO_4_·7H_2_O, 0.25 mg Na_2_MoO_4_·2H_2_O, 0.025 mg CuSO_4_·5H_2_O, and 0.025 mg CoCl_2_·6H_2_O. Seedlings were grown in a greenhouse under a 16 h/8 h (light/dark) photoperiod with a photosynthetic photon flux density of 250 μmol m^−2^ s^−1^ [52]. At the one-leaf stage, the seedlings were subjected to drought stress by treatment with a 15% polyethylene glycol 6000 (PEG 6000) solution [20]. The roots were collected at 0, 6, 12, 24, 48, and 72 h after treatment, with three biological replicates for each time point. All samples were immediately frozen in liquid nitrogen and stored at −80 °C until processed for total RNA extraction.

### 4.6. Transcriptome Data and qRT-PCR Analysis

Transcriptome data of oat under drought stress were obtained from the NCBI Sequence Read Archive (Accession number: SRP237902) [53]. Raw reads were quality-controlled with Fastp (v0.23.4) and subsequently aligned to the oat genome (sang V1.1) using HISAT2 (v2.2.1) [54]. Transcript abundance was quantified in each sample with StringTie (v2.2.3) and normalized as TPM [55]. Genes with TPM values < 0.5 across all examined tissues were considered unexpressed [56]. The expression data for the *AsGRF* gene family were then extracted and visualized as a heatmap using the Heatmap program in TBtools.

Following total RNA extraction using the DP432 Kit (Tiangen, Beijing China) and subsequent cDNA synthesis with the HiScript III 1st-Strand cDNA Synthesis Kit (Vazyme, Nanjing China). Gene expression analysis was performed by qRT-PCR using FastReal qPCR PreMix (Tiangen, China). The qRT-PCR reactions were run under the following cycling conditions: an initial step at 95 °C for 2 min, followed by 40 cycles of 95 °C for 5 s and 60 °C for 15 s. *AsActin* (GI: 226984925) was used as the endogenous control, with all primer sequences listed in Table S9. Relative expression levels were determined by the 2^−ΔΔCt^ method, and results are expressed as mean ± SEM of three technical replicates [57].

### 4.7. Subcellular Localization Analysis

The coding sequence of *AsGRF3*, excluding the stop codon, was fused in-frame to the N-terminus of green fluorescent protein (GFP) in the *pCAMBIA1302* vector. The *AsGRF3-GFP* construct, along with the empty-vector control and an *NLS-mCherry* nuclear marker, was introduced into *N. benthamiana* leaves via *Agrobacterium* (GV3101)-mediated transient transformation [58]. GFP fluorescence signals were observed using a confocal laser-scanning microscope (LSM800, Carl Zeiss, Oberkochen, Germany). The subcellular localization of the AsGRF3 protein was confirmed through three independent biological replicates.

### 4.8. Transcriptional Self-Activation Activity Analysis

The transcriptional self-activation activity of AsGRF3 was evaluated in yeast cells. The full-length coding sequence were cloned into *pGBKT7* vector, and the resulting recombinant plasmid (*pGBKT7-AsGRF3*) was transformed into the AH109 yeast strain [59]. Transformants were selected and assayed on SD/-Trp, SD/-Trp-His, and SD/-Trp-His media supplemented with X-α-Gal. The empty *pGBKT7* vector and *pGBKT7-VP16* served as negative and positive controls, respectively.

## 5. Conclusions

In this study, we systematically identified 28 *AsGRF* genes and phylogenetically classified them into two main groups comprising five subgroups. Members within the same subgroup shared highly similar exon-intron structures and conserved motif compositions. Evolutionary analysis indicated that the *AsGRF* genes have undergone strong purifying selection. Furthermore, expression profiling under drought stress highlighted *AsGRF3* as the most highly expressed member. Subcellular localization confirmed its nuclear localization, yet it lacked transcriptional self-activation activity in yeast assays. Collectively, these findings provide a foundation for elucidating the biological roles of *AsGRF* genes and their potential contribution to drought-stress adaptation.

## Figures and Tables

**Table 1:** Information about the *AsGRF* genes in oat.

Gene Name	Sequence ID	Number of Amino Acid	Molecular Weight (kDa)	pI	Instability Index	Aliphatic Index	GRAVY	QLQ Domain	WRC Domain	Predicted Subcellular Location
*AsGRF1*	AVESA.00010b.r2.2AG0234890.2	265	27.92	10.04	53.75	74.45	−0.24	103–137	169–210	Nucleus
*AsGRF2*	AVESA.00010b.r2.2AG0237710.1	401	43.88	7.73	55.90	54.44	−0.57	60–94	122–163	Nucleus
*AsGRF3*	AVESA.00010b.r2.2CG0285900.1	261	27.64	5.02	54.72	63.98	−0.64	108–142	175–216	Nucleus
*AsGRF4*	AVESA.00010b.r2.2CG0314200.1	230	24.42	10.26	51.77	71.39	−0.32	70–104	136–177	Nucleus
*AsGRF5*	AVESA.00010b.r2.2CG0316620.1	391	42.91	8.21	51.45	54.07	−0.58	55–89	117–158	Nucleus
*AsGRF6*	AVESA.00010b.r2.2DG0365750.1	401	43.92	7.76	55.76	55.89	−0.56	60–94	122–163	Nucleus
*AsGRF7*	AVESA.00010b.r2.2DG0368580.2	264	27.85	10.04	53.27	71.44	−0.30	104–138	170–211	Nucleus
*AsGRF8*	AVESA.00010b.r2.4AG0600200.1	417	46.50	8.81	61.29	59.74	−0.83	90–123	150–191	Nucleus
*AsGRF9*	AVESA.00010b.r2.4AG0604750.1	585	62.34	6.19	53.45	70.48	−0.38	141–175	208–248	Nucleus
*AsGRF10*	AVESA.00010b.r2.4DG0743940.1	420	46.73	8.81	62.00	57.00	−0.85	91–124	151–192	Nucleus
*AsGRF11*	AVESA.00010b.r2.4DG0748580.1	587	62.53	6.19	53.21	69.40	−0.39	143–177	210–250	Nucleus
*AsGRF12*	AVESA.00010b.r2.5CG0865940.1	350	38.27	8.79	62.27	51.40	−0.70	27–60	101–142	Nucleus
*AsGRF13*	AVESA.00010b.r2.5CG0871910.1	332	36.38	8.41	67.46	49.25	−0.83	19–52	93–134	Nucleus
*AsGRF14*	AVESA.00010b.r2.6AG1029740.1	408	43.58	8.64	61.52	57.30	−0.56	59–93	122–163	Nucleus
*AsGRF15*	AVESA.00010b.r2.6AG1031180.1	220	22.96	9.77	50.64	71.50	−0.26	65–98	130–171	Nucleus
*AsGRF16*	AVESA.00010b.r2.6AG1042640.1	424	45.85	7.79	49.10	49.81	−0.74	24–58	97–138	Nucleus
*AsGRF17*	AVESA.00010b.r2.6CG1083510.2	244	26.13	9.55	49.55	74.02	−0.20	98–131	163–204	Nucleus
*AsGRF18*	AVESA.00010b.r2.6CG1085280.1	398	42.60	9.13	58.32	58.49	−0.56	58–92	121–162	Nucleus
*AsGRF19*	AVESA.00010b.r2.6CG1133390.1	420	45.25	7.44	49.12	49.86	−0.74	24–58	97–138	Nucleus
*AsGRF20*	AVESA.00010b.r2.6DG1157660.1	420	45.62	7.79	48.61	49.36	−0.76	24–58	97–138	Nucleus
*AsGRF21*	AVESA.00010b.r2.6DG1168640.1	220	23.12	9.90	52.03	72.41	−0.27	65–98	130–171	Nucleus
*AsGRF22*	AVESA.00010b.r2.6DG1170080.1	403	43.16	8.44	63.38	57.77	−0.58	59–93	122–163	Nucleus
*AsGRF23*	AVESA.00010b.r2.7AG1199850.1	347	38.04	8.95	62.21	50.98	−0.71	26–59	99–140	Nucleus
*AsGRF24*	AVESA.00010b.r2.7AG1208160.1	323	35.48	8.40	66.30	47.28	−0.84	18–51	92–133	Nucleus
*AsGRF25*	AVESA.00010b.r2.7CG0695720.1	415	46.46	9.00	59.12	57.23	−0.87	88–121	148–189	Nucleus
*AsGRF26*	AVESA.00010b.r2.7CG0700670.1	588	62.71	6.16	54.19	70.12	−0.40	140–174	208–248	Nucleus
*AsGRF27*	AVESA.00010b.r2.7DG1335440.1	349	38.11	8.95	62.98	50.69	−0.70	26–59	99–140	Nucleus
*AsGRF28*	AVESA.00010b.r2.7DG1399520.1	325	35.66	8.60	68.64	46.98	−0.86	19–52	93–134	Nucleus

## Data Availability

The original contributions presented in the study are included in the article and Supplementary Material, further inquiries can be directed to the corresponding author.

## Supplementary Materials

The following supporting information can be downloaded at https://www.mdpi.com/article/10.3390/plants15010160/s1, Figure S1: The detailed information of conserved motifs in AsGRF proteins. Figure S2: Compositional profile of *cis*-regulatory elements in *AsGRF* promoters. Figure S3: Heatmap showing the expression patterns of *AsGRF* genes across root samples from RNA-seq data. Figure S4: Multiple sequence alignment of AsGRF protein sequences. Table S1: Segmentally duplicated *AsGRF* gene pairs. Table S2: The *Ka*/*Ks* ratios and distribution of duplicate *AsGRF* genes. Table S3: Orthologous relationships between *A. sativa* and *A. thaliana*. Table S4: Orthologous relationships between *A. sativa* and *O. sativa*. Table S5: Orthologous relationships between *A. sativa* and *T. aestivum*. Table S6: The expression levels of *AsGRF* genes at the transcriptome level. Table S7: *Ka*/*Ks* ratios of *GRF* orthologous genes among *A. sativa*, *O. sativa*, and *T. aestivum*. Table S8: GRF proteins from *A. thaliana*, *O. sativa*, *T. aestivum* and *A. sativa*. Table S9: Primer sequences used for *AsGRF* genes.
