# Supplementary material for: Comprehensive Investigation of GRF Transcription Factors and Associated Responses to Drought Stress in Oat (Avena sativa)
Source: Plants (Basel). 2026 Jan 5;15(1):160. doi: 10.3390/plants15010160 (PMC12787821; doi:10.3390/plants15010160)
Supplement: Supplementary file 1 [file plants-15-00160-s001.zip › no track changes mode revision-Supplementary Figure 1-4.pdf]

Supplementary Material

Supplementary Figures

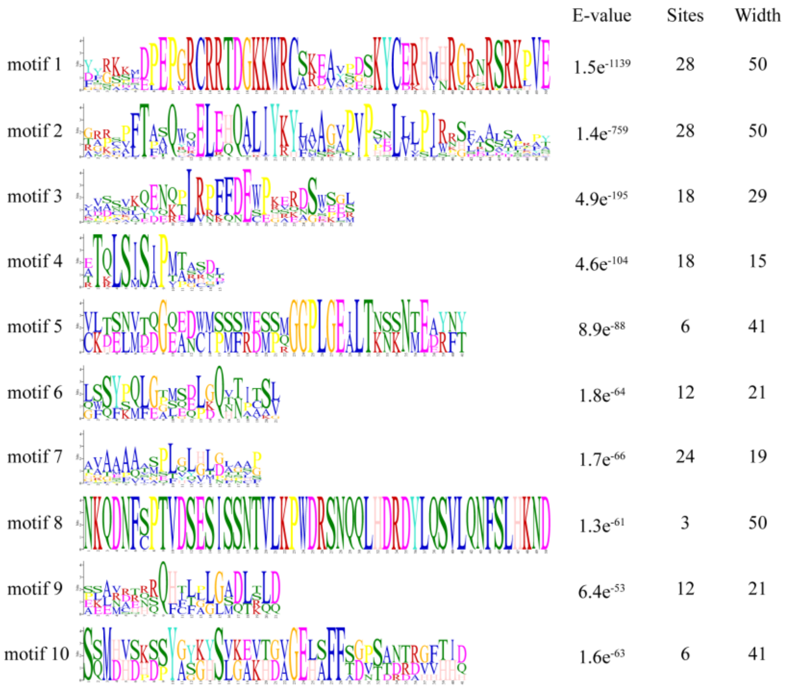

Figure S1. The detailed information of conserved motifs in AsGRF proteins.

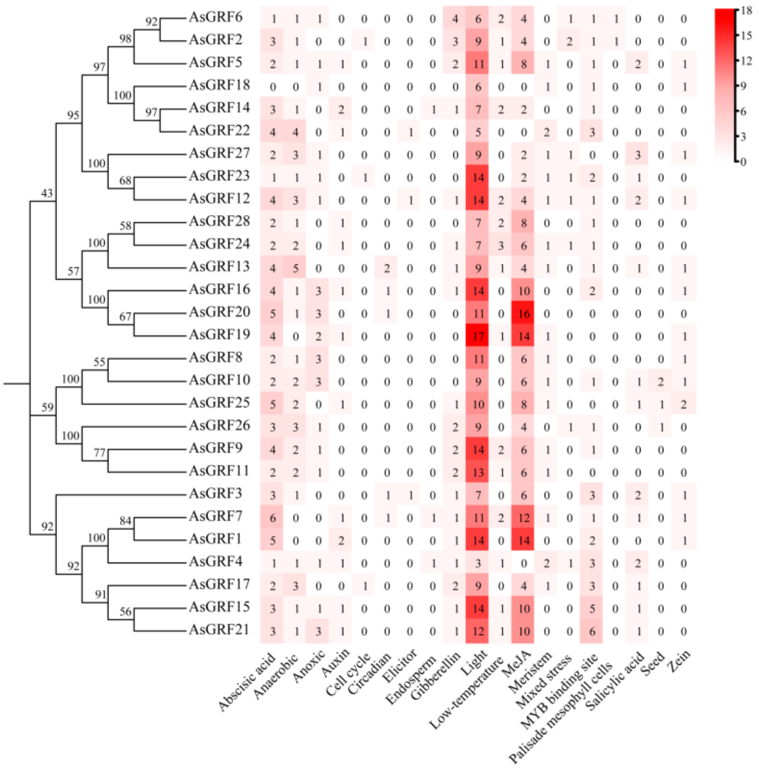

Figure S2. Compositional profile of *cis*-regulatory elements in *AsGRF* promoters.

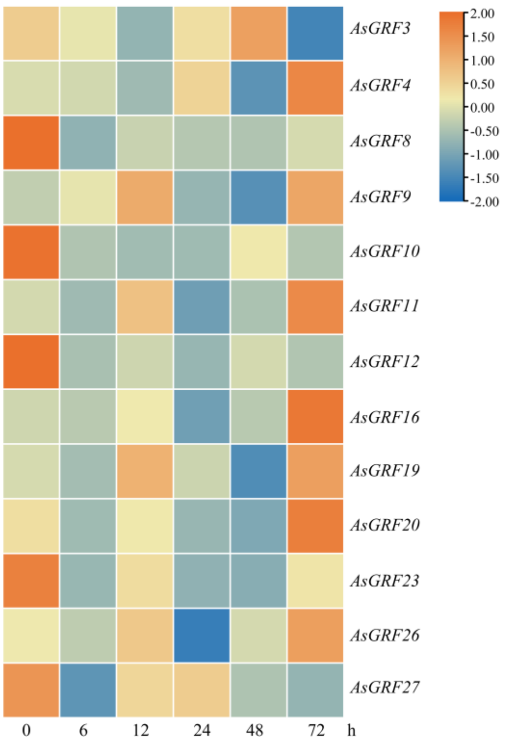

Figure S3. Heatmap showing the expression patterns of *AsGRF* genes across root samples from RNA-seq data.

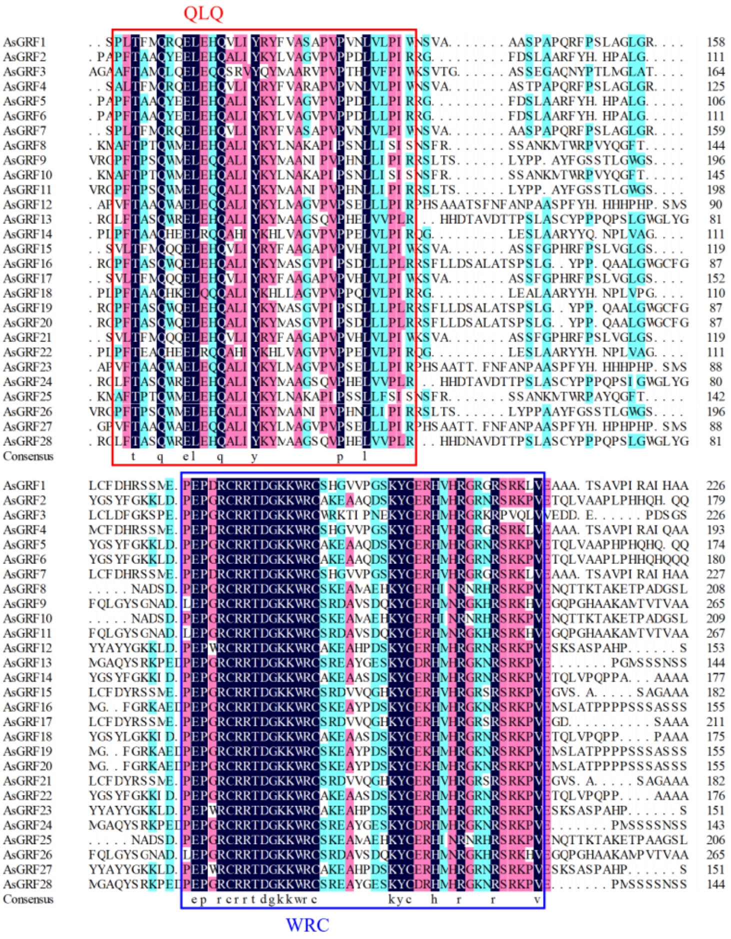

Figure S4. Multiple sequence alignment of AsGRF protein sequences.
